# Aging Alone With Dementia: Navigating Challenges and Support Needs

**DOI:** 10.1093/geroni/igaf122.882

**Published:** 2025-12-31

**Authors:** Allison Gibson, Dimitra Tziarli, Leslie McClure, Max Zubatsky, Marla Berg-Weger

**Affiliations:** Saint Louis University, Saint Louis University, Missouri, United States; Saint Louis University, St. Louis, Missouri, United States; Saint Louis University, St. Louis, Missouri, United States; Saint Lewis University, Saint Louis, Missouri, United States; Saint Louis University - - St Louis, MO, St. Louis, Missouri, United States

## Abstract

More than one-third of older adults live alone in the U.S., and the proportion increases with age. Fueling this phenomenon is a preference for the independence and privacy that living alone affords, a desire to remain in one’s private residence in later life, and long-standing sociodemographic trends. The prevalence of dementia is also steadily increasing. Aging in place, the concept of residing within one’s home as one age is a common goal for most older adults, and a supportive environment can help achieve such goals. However, the onset of dementia often derails one’s ability to age in place due to the necessity of caregiving for support and safety. This study explored the experience of persons living alone with dementia about their preferences about their memory concerns, their use of healthcare and social services, their informal social support, and their preferences for aging in place. Qualitative interviews with 15 individuals were identified through collaboration with an emergency medical services program. Themes from the interviews included challenges to medication adherence, difficulty managing finances, use of emergency services, and fear of being forced into institutionalization. While some participants had someone who was assisting them (i.e., nephew, neighbor, sibling), many participants reported they did not have any support. The majority of the participants also indicated an interest in getting additional support for their needs but felt they needed help to do so. The presentation will conclude with a discussion on potential interventions and policy implications to better support individuals living alone with dementia.

